# Rapamycin enhances neurovascular, peripheral metabolic, and immune function in cognitively normal, middle-aged APOE4 Carriers: genotype-dependent effects compared to non-carriers

**DOI:** 10.21203/rs.3.rs-6214340/v1

**Published:** 2025-03-19

**Authors:** Ai-Ling Lin, Chetan Aware, Caitlin Neher, Mohammad Hamdi, Aaron Ericsson, Oleksandr Khegai, James Patrie, Mehmet Kurt, Maalavika Govindarajan, Carter Woods, Kira Ivanich, David Beversdorf, Jianlin Cheng, Priti Balchandani, Mitzi Gonzales, Talissa Altes

**Affiliations:** University of Missouri; University of Missouri; University of Washington; University of Missouri; University of Missouri; Icahn School of Medicine at Mount Sinai; University of Virginia; University of Washington; University of Missouri; University of Missouri; University of Missouri; University of Missouri; University of Missouri; Icahn School of Medicine at Mount Sinai; Cedars Sinai Medical Center; University of Missouri

## Abstract

Rapamycin, known for its anti-aging properties, shows promise as a preventive strategy for Alzheimer’s disease (AD) in APOE4 carriers–the highest-risk group for late-onset AD. Here we show that a 4-week open-label trial of low-dose Rapamycin (Sirolimus; 1mg/day) significantly improved cerebral blood flow (CBF) relative to baseline in cognitively normal APOE4 carriers (E4(+)) aged 45–65. It also reduced inflammatory cytokines, enhanced lipid metabolism, increased short-chain fatty acids (SCFA) and enriched gut microbiome composition linked to SCFA production. Conversely, non-carriers (E4(−)) displayed stable baseline-to-post-treatment CBF and SCFA and demonstrated different treatment-related patterns of metabolic and anti-inflammatory effects than E4(+). Serum amyloid A and tau remained unchanged for both groups. These findings suggest Rapamycin may counter early vascular and metabolic deficits in E4(+) individuals, with genotype-specific effects. By bridging anti-aging research and AD prevention, this study highlights a novel, safe, and precision-based approach to mitigating AD risk in APOE4 carriers.

## INTRODUCTION

Alzheimer’s disease (AD), the most common form of dementia, is the fifth-leading cause of death among individuals aged 65 and older, affecting approximately 50 million people worldwide^1^. The apolipoprotein ε4 (APOE4) allele is the strongest genetic risk factor for late-onset AD, increasing disease risk by 3–7 times in heterozygous carriers and up to 12-fold in homozygous individuals^2,3^. Neurodegeneration associated with APOE4 begins decades before clinical symptoms appear, marked by early deficits in cerebral blood flow (CBF), impaired glucose metabolism, and gut microbiome dysbiosis^4–6^. These vascular, metabolic, and inflammatory disturbances play a critical role in cognitive decline, highlighting a key window for early intervention.

Advanced age is also a major risk factor for AD. Slowing brain aging and neurodegeneration is crucial for asymptomatic APOE4 carriers to reduce AD risk. Rapamycin is an FDA-approved medication with established anti-aging properties^7^. It is an inhibitor of mammalian target of Rapamycin (mTOR), which regulates cellular responses to nutrients, growth factors, energy status. Inhibition of mTOR has shown to extending lifespan in animal models^7,8^, enhance vascular function^9^, restore mitochondrial activity^9^, promote autophagy, and reduce amyloid-beta (Aβ) and tau deposition^10,11^. In humans, low-dose mTOR inhibition (1 mg/day or 6mg/week) has improved cognition and reduced depression^12^, and enhanced immune response during influenza vaccination in older adults^13^. These findings suggest low-dose mTOR inhibitors can be safely administered^14^, with generally mild side effects such as acneiform rash, mouth sores, and hypercholesterolemia, which are well-tolerated in healthy older adults^15^.

While clinical trials of Rapamycin are currently underway for symptomatic AD^16^, emerging preclinical data suggest that its greatest potential lies in the earliest stages of disease, where it may slow pathology progression and preserve cognitive function^17^. Preclinical studies indicate Rapamycin may counteract APOE4-driven neurodegeneration by improving CBF, brain metabolism, and reducing Aβ accumulation^17,18^, supporting its potential as a targeted AD prevention strategy.

This study represents the first in-human trial evaluating Rapamycin as a preventive intervention for AD by targeting middle-aged APOE4 carriers at elevated risk for the disease. The open-label clinical trial evaluates whether findings from APOE4 mouse models translate to humans^17^. Specifically, our goal is to determine whether a four-week, low-dose Rapamycin regimen (1 mg/day) provides similar benefits in cognitively normal, middle-aged APOE4 carriers. The primary endpoint is CBF, with secondary endpoints assessing peripheral metabolic shifts, inflammatory markers, and gut microbiome composition. An exploratory endpoint examines whether CBF responses vary by sex. Additionally, we investigate whether non-carriers exhibit distinct responses compared with APOE4 carriers, providing insights into the role of the APOE genotype in modulating Rapamycin’s effects. By integrating anti-aging research with AD prevention, this study aims to explore Rapamycin as a potential strategy to mitigate AD risk in high-risk APOE4 populations while uncovering genotype-specific responses to treatment.

## RESULTS

### Trial design and characteristics of study participants

**Figure 1:**
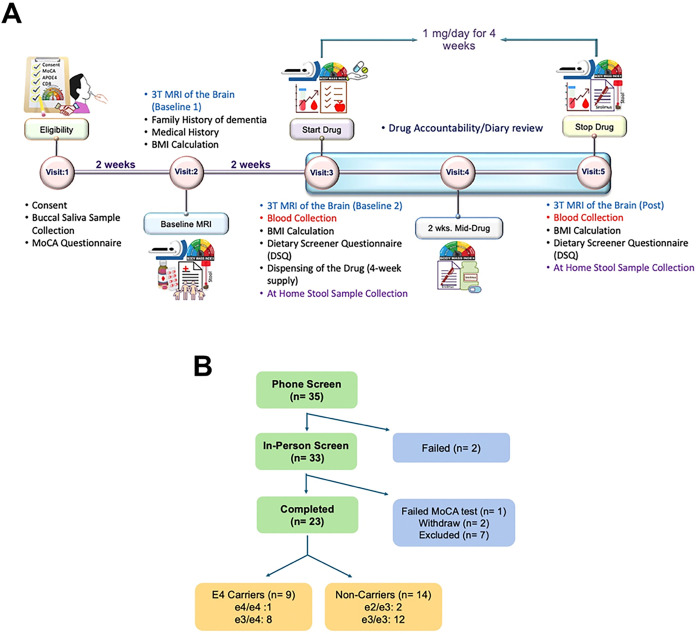
**(A)** The study design and timeline of the clinical trial.**(B)** The CONSORT diagram of participant selection, exclusions, and enrollments.

This study enrolled cognitively normal adults aged 45–65 years (MoCA ^3^ 26) without diabetes. Detailed eligibility criteria are provided in Table 1. This open-label, proof-of-concept Phase II pilot study was conducted at a single site and included five study visits over eight weeks (Fig. 1A). After providing written informed consent, candidates attended an in-person screening visit (Visit 1), which involved buccal saliva collection for APOE genotyping and cognitive screening using the Montreal Cognitive Assessment (MoCA)^19^. Once eligibility was confirmed, participants underwent a baseline assessment (Visit 2), including CBF measurement using 3T MRI, medical history review, and body mass index (BMI) assessment. Pre-intervention procedures (Visit 3) included blood collection, BMI and dietary assessments, and dispensing of Rapamycin (Sirolimus; 1 mg/day for 4 weeks). Participants were also given an at-home stool sample collection kit for baseline microbiome analysis. A second baseline MRI scan was conducted to ensure CBF measurement reproducibility. Mid-study monitoring (2 weeks; Visit 4) involved drug accountability and diary reviews. The post-intervention assessment (4 weeks; Visit 5) included a repeat 3T MRI, blood collection, BMI measurement, and dietary surveys to evaluate changes from baseline. A second stool sample collection kit was provided for post-treatment microbiome analysis. The final intervention assessment was completed four weeks after the Visit 5.

Participants were recruited from Columbia, Missouri, USA. Fig. 1B shows the Consort diagram. Of the thirty-five individuals screened by phone, two did not meet eligibility requirements. Thirty-three participants attended the in-person screening visit; one failed the MoCA screening, two withdrew after enrollment, and seven were not recruited due to an overabundance of the non-carriers (E4(−) genotype). Ultimately, 23 Caucasian participants completed the intervention between March 2, 2023, and July 19, 2024. Among them, 9 were APOE4 carriers–1 homozygous (e4/e4) and 8 heterozygous (e3/e4). The remaining 14 were non-carriers, including 12 with the e3/e3 genotype and 2 with e2/e3. Both groups had comparable age, BMI, MoCA scores, blood glucose, and HbA1c levels (Table 2). The study was registered on ClinicalTrials.gov (identifier: NCT05386914) and conducted in accordance with Good Clinical Practice (GCP) guidelines. The protocol was approved by the University of Missouri Institutional Review Board (IRB), and all participants provided written informed consent.

### Primary endpoints

**Figure 2:**
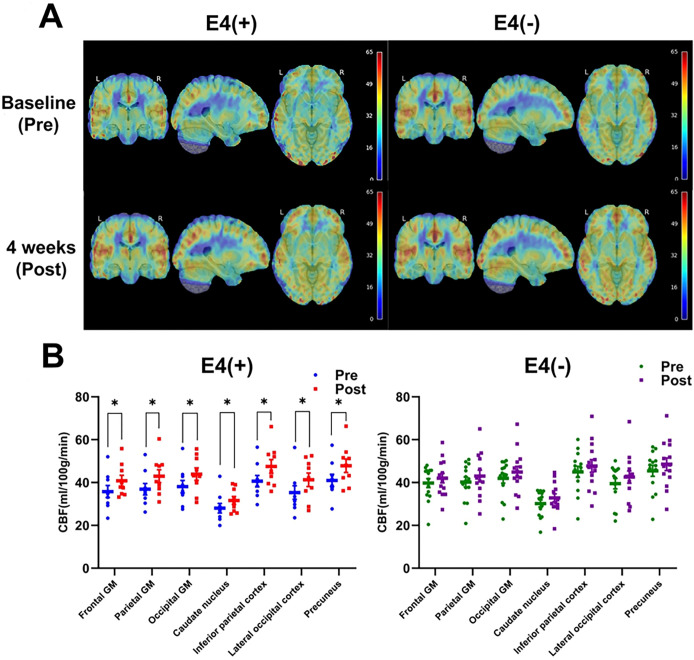
Rapamycin increases cerebral blood flow (CBF) in APOE4 carriers after 4 weeks. **(A)** CBF maps for APOE4 carriers (E4(+)) and non-carriers (E4(−)) at Baseline (Pre) and 4 weeks after the intervention (Post). The color bar represents CBF values (0–65 ml/100g/min) on a linear scale. **(B)** Regional CBF values (in ml/100g/min) at Baseline (Pre) and 4-week (Post) for E4(+) and E4(−) groups. Data are shown as mean ± SEM in scatter dot plots. Paired t-tests were used for statistical analysis. *p < 0.05. GM: Gray matter.

The primary objective of this study was to assess CBF using 3T MRI. Results indicate that the Rapamycin regimen significantly increased CBF in APOE4 carriers (E4(+)). Fig. 2A displays CBF maps for both E4(+) and E4(−) before and after the intervention, with the color bar representing CBF values from 0 to 65 ml/100g/min on a linear scale. The baseline data were averaged from two pre-treatment scans, demonstrating strong reproducibility with a variance of less than 1% between them. At baseline, E4(+) participants exhibited a trend toward lower global CBF compared to E4(−) (33.6 ± 5.4 vs. 36.8 ± 7.2 ml/100g/min, respectively), though this difference was not statistically significant (p > 0.05). Paired t-tests comparing pre- and post-treatment CBF values (Fig. 2B) showed a significant (>15%, p < 0.05) increase in CBF across multiple brain regions in E4(+) individuals. Notable areas included gray matter (GM) in the frontal, parietal, and occipital lobes, as well as the caudate nucleus, inferior parietal cortex, lateral occipital cortex, and precuneus–regions essential for cognitive function. On the other hand, no significant CBF changes were observed in the E4(−) group.

### Secondary endpoints

The secondary aims of the trial were to assess baseline-to-post-treatment changes in (i) plasma metabolomic and short-chain fatty acids (SCFA) profiles, (ii) plasma markers associated with AD pathology, (iii) plasma cytokine levels, and (iv) gut microbiome diversity and composition.

#### Plasma metabolomics and SCFA

**Figure 3:**
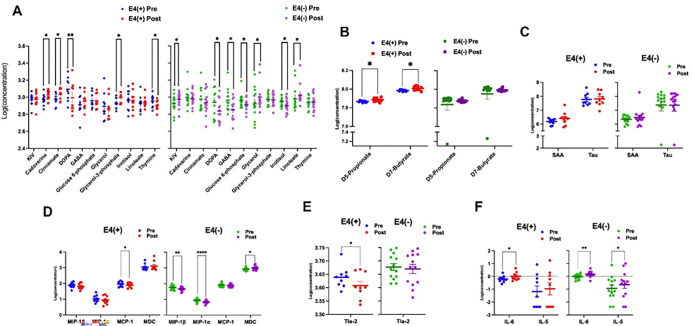
Plasma metabolomics, SCFA, AD biomarkers and inflammatory cytokines. **(A)** Scatter dot plots illustrate changes in Loge(metabolite) concentrations before (Pre) and after (Post) Rapamycin intervention in the APOE4 carriers (E4(+)) and non-carriers (E4(−)) groups. **(B)** Log-transformed level of short-chain-fatty-acid (SCFA) before and after intervention for both groups. **(C)** Log-transformed level of plasma SAA and Tau - Alzheimer’s disease biomarkers – in E4(+) and E4(−) groups pre- and post-intervention. (**D-F**) Log-transformed level of various inflammatory cytokines before and after intervention for both groups. MIP-1α: Macrophage inflammatory protein-1 alpha; MIP-1β: macrophage inflammatory protein-1 beta; MCP-1: monocyte chemoattractant protein-1; MDC: macrophage-derived chemokine; Tle-2: thymic leukemia antigen-2; IL-6; interleukin-6; IL-5 interleukin-5. All charts present mean ± SEM. Paired t-test was used to determine p-values, with *p < 0.05; **p < 0.01; ****p < 0.0001.

Plasma samples were analyzed by non-targeted metabolomics and SCFA panels. In the E4(+) group, significant post-treatment increases were observed in Cadaverine, a polyamine involved in cellular signaling and microbial processes; Cinnamate, a precursor for antioxidant; and Glycerol-3-phosphate (G3P), a critical metabolite in glycolysis and lipid synthesis (Fig. 3A). On the other hand, decreases were noted in DOPA (3,4-dihydroxyphenylalanine), a precursor to dopamine and other catecholamines, and Thymine, a DNA nucleobase crucial for genetic stability. Similarly, the E4(−) group exhibited decreased DOPA levels, along with reduced levels of Gamma-Aminobutyric Acid (GABA), a key inhibitory neurotransmitter, Glucose-6-phosphate (G6P), an intermediate in glycolysis and the pentose phosphate pathway, and Inositol, essential for cell signaling and membrane stability. The E4(−) group also showed increased levels of Linoleate, an omega-6 fatty acid involved in lipid signaling, Glycerol, a substrate for energy production and lipid synthesis, and Keto-isovalerate (KIV), a branched-chain amino acid intermediate linked to energy metabolism.

Significant changes in SCFA were observed in the E4(+) group, including increases in *D7-Butyrate* and *D5-Propionate*, both of which are beneficial SCFAs known to support gut health and immune regulation (Fig. 3B). In contrast, these SCFA changes were not detected in the E4(−) group.

#### AD biomarkers

Baseline (pre-treatment) and post-treatment levels of plasma serum amyloid A (SAA) and phosphorylated Tau (pT217) were assessed using the MesoScale Discovery (MSD) chemiluminescence-based assays. SAA and pT217 showed no significant differences between the E4(+) and E4(−) groups at the baseline (p > 0.05). No significant changes were observed in post-treatment in either SAA or pT217 levels in both E4(+) and E4(−) individuals (p > 0.05) (Fig. 3C).

#### Plasma cytokine levels

Plasma levels of 30 inflammatory biomarkers were assessed using MSD assays. Fig. 3D shows that the E4(+) group experienced significant post-treatment reductions in monocyte chemoattractant protein-1 (MCP-1), a key regulator of inflammation and tissue damage. Conversely, the E4(−) group showed significant decreases in macrophage inflammatory protein-1-beta (MIP-1β) and macrophage inflammatory protein-1-alpha (MIP-1α), along with an increase in macrophage-derived chemokine (MDC). Fig. 3E presents a decrease in Tle-2, a key angiogenesis marker and tyrosine kinase with immunoglobulin and EGF homology domains, in the E4(+) group, but not in the E4(−) group. Fig. 3F highlights significant increases in interleukin-6 (IL-6), a major pro-inflammatory cytokine linked to systemic inflammation, in both groups. However, interleukin-5 (IL-5), which plays a role in anti-inflammatory and allergic responses, increased only in the E4(−) group.

#### Gut microbiome

**Figure 4:**
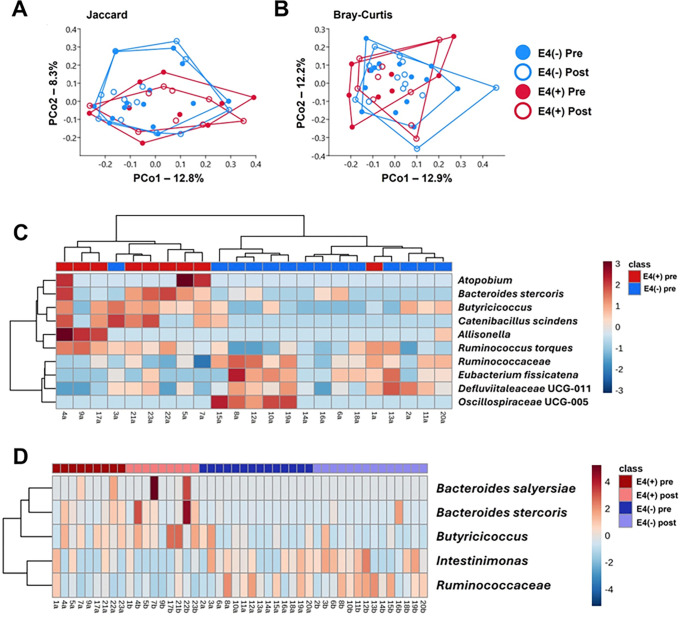
Gut Microbiome Composition Differences in APOE4 Carriers and Non-Carriers. **(A)** Principal coordinate analysis (PCoA) plot based on unweighted Jaccard dissimilarities, illustrating differences in fecal microbiome composition between APOE4 carriers (E4(+)) and non-carriers (E4(−)) at baseline and endpoint. Statistical results from two-way PERMANOVA: E4 status (p = 0.0076, F = 1.6), Timepoint (p = 1, F = 0.3), Interaction (p = 0.54, F = −3.0). **(B)** PCoA plot based on weighted Bray-Curtis dissimilarities, showing microbial community shifts with statistical results from two-way PERMANOVA: E4 status (p = 0.322, F = 1.0), Timepoint (p = 1, F = 0.4), Interaction (p = 0.76, F = −3.4). **(C)** Heatmap of hierarchical clustering based on the relative abundance of the top 10 amplicon sequence variants (ASVs) with raw p-values < 0.05 in serial Kruskal-Wallis ANOVA on ranks. **(D)** Hierarchical clustering of the relative abundance of the five ASVs that showed significant differences (raw p-values < 0.05) in Kruskal-Wallis testing. Subject groups are indicated in the color bar at the top, with the legend displayed on the right.

For gut microbiome, principal coordinate analysis (PCoA) of samples from both groups at both time points indicated partial overlap based on APOE4 status, with minimal influence of time. A two-way permutational multivariate analysis of variance (PERMANOVA) on Jaccard dissimilarities showed a significant effect of APOE4 status (p = 0.0076, F = 1.6), while time and interaction effects were not significant (Fig. 4A). However, PERMANOVA based on Bray-Curtis dissimilarities did not detect significant effects of APOE4 status or time (Fig. 4B). Since Jaccard dissimilarities emphasize taxa presence/absence–often at low relative abundance (RA)–while Bray-Curtis prioritizes differences in higher RA taxa, these findings suggest that sustained differences in the fecal microbiome of E4(+) are primarily driven by rare taxa.

Hierarchical clustering of Amplicon Sequence Variant (ASV) relative abundance showed a near-complete separation between E4(+) and E4(−) groups at baseline (pre-treatment) (Fig. 4C). Specifically, E4(+) individuals exhibited higher levels of *Atopobium, Bacteroides stercoris, Butyricicoccus, Catenibacillus scindens, Allisonella*, and *Ruminococcus torques*, whereas E4(−) individuals had greater abundances of *Ruminococcaceae, Eubacterium fissicatena, Defluviitaleaceae UCG-011*, and *Oscillospiraceae UCG-005*. To identify post-treatment ASVs contributing to these differences, serial Kruskal-Wallis tests were performed. Although no ASVs remained significant after multiple testing correction, five exhibited raw p-values <0.05. Three ASVs (*Bacteroides salyersiae, Bacteroides stercoris*, and *Butyricicoccus sp*.) were enriched in E4(+), but largely absent in E4(−) (Fig. 4D). Conversely, ASVs corresponding to *Intestinimonas sp*. and an unclassified *Ruminococcaceae* genus were more abundant in E4(−). Overall, fecal microbiome analysis indicated distinct APOE4-associated differences, primarily driven by the presence or absence of low-abundance taxa, with specific bacterial species enriched or depleted based on APOE4 carrier status.

### Exploratory endpoints

Given the high spatial resolution and sensitivity of CBF measurements at an individual level using MRI, we were able to explore sex-stratified analyses despite the small sample size. **Supplementary Figure 1** illustrates baseline CBF and its changes following four weeks of Rapamycin treatment across APOE4 genotypes and sexes. At baseline, E4(−) individuals exhibited higher brain perfusion compared to E4(+), with female E4(+) displaying the lowest CBF among all groups, though this difference did not reach statistical significance (**Supp Fig. 1A**). After four weeks of Rapamycin treatment, CBF changes were observed in a genotype- and sex-dependent manner, with female E4(+) showing the most pronounced increases in perfusion across the entire brain (**Supp Fig. 1B**).

Quantitative analysis of percentage CBF changes in key brain regions associated with cognition showed significant perfusion improvements in female E4(+) participants. Specifically, CBF increased by approximately 30% in the hippocampus, 35% in the cortex, and 35% in the whole brain. In contrast, male E4(+) participants and both male and female E4(−) groups exhibited negligible responses to Rapamycin, with no significant changes detected in these regions (**Supp Fig. 1C**). To further examine region-specific CBF changes, we conducted a heatmap analysis, which highlighted substantial increases across multiple brain areas in female E4(+). Most regions exhibited CBF increases exceeding 20% after four weeks of Rapamycin administration, with the inferior temporal gyrus showing the most pronounced improvement (~45%). However, no significant changes were observed in male E4(+) or both E4(−) groups (p > 0.05) (**Supp Fig. 1D**).

### Safety and adherence

Administering Rapamycin at a dose of 1 mg per day for 4 weeks maintained hematological parameters well within the normal range, with only subtle fluctuations. Detailed hematological data are presented in Table 3. The study confirmed the safety and tolerability of Rapamycin treatment, with no clinically significant adverse effects. Both E4(+) and E4(−) groups demonstrated stable BMI and no notable changes in liver or kidney function markers, apart from a minor but significant increase in alkaline phosphatase levels in the E4(−) group (p = 0.0445). Hematological parameters revealed reductions in red cell distribution width (RDW) in both groups, indicating decreased erythrocyte size variability, while hemoglobin and platelet counts remained stable. Immune modulation was evident, with a reduction in % immature granulocytes and absolute lymphocyte counts in the E4(+) group and a decrease in absolute basophil counts in both groups. Electrolytes remained stable overall, with slight decreases in potassium and anion gap noted in the E4− group. Lipid and glucose metabolism markers, including triglycerides and HbA1c, remained within clinically acceptable ranges, though a small HbA1c increase was observed in E4(+) individuals (p = 0.0168). No major side effects were reported, except two reported loose stool, and one reported runny nose, stomachache, and swollen eyelid. These findings demonstrate the tolerability of Rapamycin and highlight its potential for genotype-specific metabolic and immune modulation in precision medicine applications.

## DISCUSSION

For the first time, we demonstrated that Rapamycin enhances neurovascular function, modulates peripheral metabolism, improves immune responses, and enriches microbiome composition in middle-aged, cognitively normal APOE4 carriers. Table 4 summarizes the key findings of our study. Briefly, with Rapamycin, E4(+) individuals (particularly females) exhibited a significant increase in CBF in brain regions crucial for cognitive function that display early alterations in the preclinical stage of AD, including the frontal and parietal lobes, caudate nucleus, and precuneus. Plasma metabolomics analysis demonstrated decreased thymine and DOPA levels, alongside elevated levels of G3P, cinnamate, and cadaverine. Immunological changes included reduced MCP-1 and Tle-2 levels, increased IL-6, and elevated SCFA, particularly butyrate and propionate. These SCFA changes aligned with gut microbiome alterations, where Rapamycin enriched *Bacteroides salyersiae, Bacteroides stercoris*, and *Butyricicoccus sp*. in E4(+) individuals–microbes known for their SCFA synthesis capabilities. Hematological analysis showed stable blood parameters, except for a reduction in RDW after four weeks of Rapamycin treatment. Notably, serum amyloid A (SAA/Aβ) and tau levels remained unchanged throughout the trial.

These findings highlight the potential of Rapamycin as an early intervention for APOE4 carriers at risk for AD. Impaired CBF is a major risk factor for AD, with women facing a disproportionately higher risk than men^20^. Enhancing CBF while individuals are still cognitively normal may provide neuroprotection and help mitigate the risk of late-onset AD, particularly in E4(+) females. This finding aligns with our preclinical studies, where female E4(+) mice exhibited the strongest response to Rapamycin compared to other groups^17^. The differential effects of Rapamycin between sexes may be attributed to distinct regulation of the mTOR pathway, which is influenced by sex hormones such as estrogen and testosterone^21^. Estrogen, in particular, has vasodilatory effects that enhance blood flow through endothelial nitric oxide production, a mechanism linked to mTOR inhibition^22^. Furthermore, sex-specific utilization of mTOR signaling branches suggests that hormonal differences contribute to variations in mTOR pathway regulation. This aligns with previous findings that female mice often experience a more pronounced lifespan extension from Rapamycin treatment compared to males^23^, likely due to sex-based metabolic and immune differences influencing Rapamycin’s effectiveness. Moreover, several E4(+) participants in this trial reported subjective improvements in mood, energy levels, and sleep quality after taking Rapamycin. These anecdotal reports align with existing literature suggesting that Rapamycin may alleviate depression^12^, potentially through its effects on CBF enhancement and improved brain circulation^24,25^. Future studies using validated questionnaires will be necessary to further evaluate Rapamycin’ effect on these outcomes.

An intriguing finding in this study is the reduction in RDW following Rapamycin treatment in both E4(+) and E4(−) groups. RDW reflects the variability in the size of circulating red blood cells (anisocytosis) and is traditionally used in clinical hematology to assess conditions like anemia. Emerging evidence suggests that RDW is increasingly recognized as a marker of biological aging, chronic inflammation, and oxidative stress^26^. The observed reduction in RDW may support the idea that Rapamycin, as an mTOR inhibitor, can slow down aging-related processes. Interestingly, this finding aligns with previous research showing that, in midlife, RDW and CBF exhibit different patterns in APOE4 carriers and non-carriers^27^. This suggests that E4(+) may have a distinct hemodynamic response to changes in red blood cell health. Our data further support this connection, showing that Rapamycin not only restored CBF but also reduced RDW specifically in the E4(+) group, reinforcing its potential as a precision intervention for vascular and metabolic aging in this high-risk group.

Rapamycin modulates key metabolic pathways in E4(+) individuals, increasing cadaverine, cinnamate, and G3P while reducing thymine levels. The rise in G3P suggests a metabolic shift toward lipid metabolism and increased neuronal energy support^28,29^. Cadaverine, a microbial metabolite, indicates alterations in the gut microbiome, accompanied by changes in SCFA profiles^30^. This aligns with our findings of increased butyrate and propionate in E4(+) participants. Cinnamate, an antioxidant, may reflect a response to oxidative stress and enhanced immune function; has been shown to suppress pro-inflammatory cytokines and mitigate oxidative stress, which corresponds with observed shifts in inflammatory markers^31,32^. In E4(+) individuals, we noted reduced MCP-1 and Tle-2 alongside elevated IL-6, suggesting a pro-inflammatory shift despite decreased chemokine signaling. The increase in cinnamate may help counterbalance excessive inflammation through its immunomodulatory properties, contributing to a more regulated immune response. Notably, the reduction in Tle-2–a factor associated with angiogenesis in cancer^33^–aligns with the protective vascular effects of mTOR inhibition.

These metabolic, inflammatory, and microbiome shifts align with mTOR inhibition effects, which reduce lipogenesis, mitochondrial biogenesis, and pyrimidine production^34–36^, and modulate inflammatory pathways^37^. Notably, the decrease in thymine may result from mTOR inhibition’s suppression of de novo pyrimidine synthesis. This lower thymine availability slows DNA synthesis and cellular proliferation, which is linked to enhanced genomic stability, reduced metabolic stress, and extended lifespan–all hallmarks of longevity-promoting pathways.

We also observed that Rapamycin elicits distinct metabolic, inflammatory, cerebrovascular, and microbiome responses in E4(−) individuals, as summarized in Table 4. Global CBF remained stable, with regional increases observed in males, suggesting a potential sex-specific cerebrovascular response to mTOR inhibition. Metabolically, E4(−) individuals exhibited increased α-KIV, glycerol, and linoleate, indicating enhanced amino acid metabolism, lipid turnover, and fatty acid metabolism, while decreased GABA, DOPA, G6P, and inositol suggest alterations in neurotransmitter synthesis and reduced glucose utilization. These findings align with our previous reports that APOE3 mice under Rapamycin treatment exhibited lower glycolysis and altered GABA levels^9,17^, reinforcing the idea that mTOR inhibition may shift E4(−) individuals toward lipid metabolism.

Inflammatory marker analysis in the E4(−) revealed increased IL-5, IL-6, and MDC, alongside decreased MIP-1α and MIP-1β, suggesting a more anti-inflammatory immune shift with modulated chemokine signaling and reduced leukocyte recruitment. This pattern implies that Rapamycin may promote a more balanced immune response in E4(−) individuals, potentially reducing chronic inflammation without excessive immune suppression. Microbiome analysis showed that E4(−) individuals did not exhibit significant SCFA changes but had an enrichment of *Intestinimonas sp*. and an unclassified *Ruminococcaceae genus*, suggesting that Rapamycin may selectively enhance beneficial gut microbial populations without drastically altering SCFA metabolism in this genotype. These results emphasize the importance of APOE-tailored approaches in Alzheimer’s prevention, with E4(−) individuals potentially benefiting from mTOR inhibition through metabolic adaptations, neuroprotection, and immune modulation rather than CBF and microbiome changes.

This study aligns with a recent *Nature Medicine* commentary emphasizing that disease prevention strategies hold great promise for addressing the growing incidence of dementia, though more clinical trial evidence is needed to confirm their feasibility and effectiveness^38^. This is particularly relevant for APOE4 carriers, who face a higher risk of developing AD later in life. The widespread availability of direct-to-consumer genetic testing has made it easier for individuals to determine their APOE status. Narasimhan et al. highlight the pivotal role of APOE4 in AD progression and highlight emerging APOE-targeted therapies that could revolutionize clinical care^39^. They advocate for incorporating APOE genotype testing into routine practice to enable personalized treatment approaches. However, they also caution that simply knowing one’s genetic risk–without available interventions–may heighten stress and anxiety. The findings from this study provide promising preventive strategies for middle-aged, presymptomatic APOE4 carriers.

We recognize that the small sample size is a limitation of this study. While the high spatial resolution and sensitivity of MRI allow for the detection of individual-level CBF changes, even in a relatively small cohort, metabolomic and microbiome analyses require larger sample sizes. These analyses involve complex and dynamic biological networks with significant inter-individual variability, making robust sex-stratified analyses more challenging. Another limitation is the lack of racial and ethnic diversity, as our study population consisted exclusively of Caucasian participants, reflecting the regional demographics of Columbia, MO, USA. We recognize the importance of diversity in capturing broader biological and clinical implications and aim to expand recruitment efforts to include more diverse populations in future trials.

Additionally, a longer study duration with a placebo-controlled design is needed to determine whether Rapamycin intervention can also preserve cognitive function in E4(+) individuals. Future research should explore these aspects to strengthen the translational potential of our findings.

In conclusion, our findings suggest that an anti-aging intervention may help mitigate AD risk in cognitively normal, middle-aged APOE4 carriers. Specifically, Rapamycin shows potential as a preventive strategy by enhancing neurovascular function, modulating peripheral metabolism, improving immune responses, and enriching microbiome composition. Moreover, we demonstrate that Rapamycin’ effects are APOE genotype-dependent, with E4(−) and E4(+) individuals benefiting through distinct mechanisms and pathways. These findings imply the importance of a precision medicine approach in developing targeted AD prevention strategies.

## ONLINE METHODS

### APOE Genotyping

Genomic DNA was extracted from buccal swabs using the Maxwell RSC Cultured Cells Kit (Cat# AS1620) on a Maxwell RSC48 Instrument (Promega). APOE genotyping was performed via PCR amplification using an M13-tailed assay targeting the region of interest (Hs00404451_CE; Thermo Cat# A15633 and A15634) in 10 μL reactions within 96-well plates, utilizing repliQa HiFi ToughMix (Quantabio). The amplicon length was 585 bp. PCR conditions included an initial denaturation at 98°C for 2 minutes, followed by 32 cycles of 98°C for 10 sec, 60°C for 1 sec, and 68°C for 1 sec. PCR products were cleaned using ExoSAP-IT^™^ Express PCR Product Cleanup (Thermo Cat# 7501.1EA) before sequencing with primer M13F on an ABI 3730xI DNA Analyzer. APOE genotypes were determined using CLC Genomics Workbench (v22; Qiagen). DNA extraction, PCR, and genotype calling were conducted at the Genomics and Microbiome Core Facility (GMCF; Rush University), while capillary electrophoresis sequencing was performed at the Genomics Research Core (GRC; University of Illinois at Chicago).

### Brain MRI Acquisition and Processing

All MRI scans were conducted at the University of Missouri on a 3T Vida scanner (Siemens Healthineers) using a Head/Neck 20 MR Coil. High resolution (1×1×1 mm^3^) structural images were acquired with a 3D T1-weighted magnetization-prepared rapid gradient-echo imaging sequence (MPRAGE, TR = 2300 ms, TE = 2.97 ms). A pseudo-continuous arterial spin-labeling (pCASL) sequence with background suppression was used for quantitative perfusion imaging reconstructed at 1.7×1.7×4.0 mm^3^ resolution; 16 volumes (8 control and 8 labeled) were acquired. The labeling duration, t_L_ = 1.8 s was followed by a single post-labeling delay of t_P_ = 1.8 s before a 3D GRASE readout with full brain coverage (TR = 4 s, TE = 22.1 ms, Matrix size = 64×64×32, FOV=220 mm, GRAPPA = 2). In addition to the T1 and pCASL data, a fully relaxed proton-density image (M0) was acquired at the same resolution as pCASL (TR = 10 s, TE = 22.1 ms, Matrix size = 64×64×32). The BASIL toolbox^40^, a research tool included in the FMRIB Software Library (FSL) was used to obtain quantified perfusion maps from the pCASL magnitude images. ASL data were processed in the native acquisition voxel space and motion correction was applied. A fast Variational Bayesian inference algorithm performed iterative nonlinear kinetic model fitting to estimate the hemodynamic parameters of CBF. Voxelwise calibration was done using the proton density-weighted MO image to estimate the magnetization of arterial blood for each voxel in the perfusion image. Alongside the perfusion analysis, volumetric brain segmentation of T1-weighted images was done using FreeSurfer v7^41,42^ and binary region of interest (ROI) masks were generated. For statistical regional analysis, CBF maps were coregistered to the T1 space by affine transformation using SPM12^43^. Mean and standard deviation values of perfusion for each ROI (of each subject, at each time point) were obtained. For group-wise visualization and statistical mapping, T1 volumes and CBF maps were warped to the MNI152 brain atlas through affine and nonlinear transformation in SPM12^43^, bringing all subject data into the same matrix space.

### Stool Sample Collection and Gut Microbiome Analysis

Participants collect stool samples were at baseline and post-imaging sessions for gut microbiome analysis. A mail-in box Blood samples were collected during imaging visits for comprehensive metabolic, inflammatory, and Rapamycin level panels. Stool samples were kept frozen until DNA extraction could be performed.

#### • Fecal DNA extraction

DNA was extracted using QIAamp PowerFecal Pro DNA extraction kits (Qiagen) according to manufacturer instructions, with the exception that samples were homogenized in bead tubes using a TissueLyser II (Qiagen) for ten minutes at 30/sec before proceeding according to the protocol and eluting in 100 μL of elution buffer. DNA yields were quantified using Qubit 2.0 (Invitrogen) and quant-iT BR dsDNA reagent kits (Invitrogen) and normalized to a uniform concentration and volume.

#### • 16S rRNA library preparation and sequencing

Library preparation and sequencing were performed at the University of Missouri (MU) Genomics Technology Core. Bacterial 16S rRNA amplicons were constructed via amplification of the V4 region of the 16 SRNA gene with universal primers (U515F/806R), flanked by Illumina standard adapter sequences^44,45^. Dual-indexed forward and reverse primers were used in all reactions. PCR was performed in 50 μL reactions containing 100 ng metagenomic DNA, primers (0.2 μM each), dNTPs (200 μM each), and Phusion high-fidelity DNA polymerase (1U, Thermo Fisher). Reaction parameters were 98°C^(3 min)^ + [98°C^(15 sec)^ + 50°C^(30 sec)^ + 72°C^(30 sec)^] × 25 cycles +72°C^(7 min)^. Amplicon pools (5 μL/ reaction) were combined, mixed, and then purified by addition of Axygen Axyprep MagPCR clean-up beads to an equal volume of 50 μL of amplicons and incubated for 15 minutes at room temperature. Products were then washed multiple times with 80% ethanol and the dried pellet was resuspended in 32.5 μL EB buffer (Qiagen), incubated for two minutes at room temperature, and then placed on the magnetic stand for five minutes. The final amplicon pool was evaluated using the Advanced Analytical Fragment Analyzer automated electrophoresis system, quantified using quant-iT HS dsDNA reagent kits, and diluted according to Illumina standard protocol for sequencing as 2×250 bp paired-end reads on the MiSeq instrument.

#### • Microbiome informatics analysis

DNA sequences were assembled and annotated at the MU Bioinformatics and Analytics Core. Primers were designed to match the 5’ ends of the forward and reverse reads. Cutadapt^46^ (version 2.6) was used to remove the primer from the 5’ end of the forward read. If found, the reverse complement of the primer to the reverse read was then removed from the forward read as were all bases downstream. Thus, a forward read could be trimmed at both ends if the insert was shorter than the amplicon length. The same approach was used on the reverse read, but with the primers in the opposite roles. Read pairs were rejected if one read or the other did not match a 5’ primer, and an error-rate of 0.1 was allowed. Two passes were made over each read to ensure removal of the second primer. A minimal overlap of three bp with the 3’ end of the primer sequence was required for removal. The QIIME2^47^ DADA2^47^ plugin (version 1.10.0) was used to denoise, de-replicate, and count ASVs (amplicon sequence variants), incorporating the following parameters: 1) forward and reverse reads were truncated to 150 bases, 2) forward and reverse reads with number of expected errors higher than 2.0 were discarded, and 3) Chimeras were detected using the consensus method and removed. R version 3.5.1 and Biom version 2.1.7 were used in QIIME2. Taxonomies were assigned to final sequences using the Silva.v132^48^ database, using the classify-sklearn procedure. Principal coordinate analysis and permutational multivariate analysis of variance (PERMANOVA) were used to visualize and test for differences in beta-diversity using PAST 4.03 software^49^. Hierarchical clustering was performed using unweighted pair group method with arithmetic mean (UPGMA) of cube root-normalized relative abundance data in MetaboAnalyst 3.0^50^. Differential change in beta-diversity and baseline relative abundance were tested using Mann-Whitney rank sum test or Kruskal Wallis test respectively.

### Complete blood count (CBC) measurements

The CBC outcomes shown in the Table 3 were run through the Complete Metabolic Panel by MU Health Care Laboratory.

### Rapamycin Concentration Measurement

1 mL Whole Blood EDTA was sent to Mayo Clinic Laboratories in Rochester for Rapamycin concentration determination. The resulting supernatant is analyzed by liquid chromatography tandem mass spectrometry (LC-MS/MS)^51^.

### Plasma Extraction and Derivatization

To extract metabolites, 80 plasma samples were diluted into 720 uL of ice-cold 2:2:1 methanol/acetonitrile/water containing a mixture of internal standards (D4-citric acid, D4-succinic acid, D8-valine, and U13C-labeled glutamine, glutamic acid, lysine, methionine, serine, and tryptophan; Cambridge Isotope Laboratories), where the 1-part water was composed of the sample volume + water. An additional deuterated Short Chain Fatty Acid standard and was also added for signal normalization. Plasma extraction mixtures were vortexed for 10 minutes at RT and rotated for 1 hour at −20°C. Mixtures were centrifuged for 10 minutes at 21,000 × g, and 150 μl of the cleared metabolite extracts were transferred to autosampler vials and dried using a SpeedVac vacuum concentrator (Thermo). Dried metabolite extracts were reconstituted in 30 μl of 11.4 mg/ml methoxyamine (MOX) in anhydrous pyridine, vortexed for 5 minutes, and heated for 1 hour at 60°C. Next, to each sample 20 μl of N,O-Bis(trimethylsilyl)trifluoroacetamide (TMS) was added, samples were vortexed for 1 minute, and heated for 30 minutes at 60°C.

### Plasma Metabolomics

Derivatized samples were analyzed by GC-MS. 1 μl of derivatized sample was injected into a Trace 1300 GC (Thermo) fitted with a TraceGold TG-5SilMS column (Thermo) operating under the following conditions: split ratio = 20:1, split flow = 24 μl/minute, purge flow = 5 ml/minute, carrier mode = Constant Flow, and carrier flow rate = 1.2 ml/minute. The GC oven temperature gradient was as follows: 80°C for 3 minutes, increasing at a rate of 20°C/minute to 280°C, and holding at a temperature at 280°C for 8 minutes. Ion detection was performed by an ISQ 7000 mass spectrometer (Thermo) operated from 3.90 to 21.00 minutes in El mode (−70 eV) using select ion monitoring (SIM). LC-MS data was acquired on a Thermo Q Exactive hybrid quadrupole Orbitrap mass spectrometer with a Vanquish Flex UHPLC system or Vanquish Horizon UHPLC system. The LC column used was a ZIC-pHILIC column (50 X 2.1 mm). The injection volume was 1μL for cecal and 10ul for serum samples of cleared metabolite extract. The method was run at a flow rate of 0.3 mL/min. The gradient starts at 80% B and decreasing to 20% B over 3.3 minutes; returning to 80% B in 0.12 minutes; and held there for 2.5 minutes. The mass spectrometer was operated in negative full-scan mode from 0.05 to 4.55 minutes.

### Plasma Multiplex Analysis of Immunomodulatory Factors with MSD Assays

Plasma immunomodulatory molecules, including cytokines, chemokines, vascular injury factors, angiogenic markers, and Tau(pT217), were quantified through chemiluminescence-based assays (Meso Scale Discovery (MSD), Rockville, Maryland, USA). Specific panels from MSD were employed for each molecule type: the V-PLEX Proinflammatory Panel-1 (catalog # K15049D-1) for proinflammatory cytokines, the V-PLEX Cytokine Panel-1 (catalog # K15050D-1) for a broader cytokine assessment, the V-PLEX Vascular Injury Panel 2 (catalog # K15198D-1) for vascular injury markers and the V-PLEX Chemokine Panel-1 (human) Generation B (catalog # K15705D-1) for chemokines, Tau (pT217) levels were determined using the S-PLEX Human Tau (pT217) Kit (catalog # K151APFS-1), and For angiogenic factors, the V-PLEX Custom Human Biomarkers-1 (catalog # K151ARH-1) panel was utilized. The lower limits of detection (LLOD) for the assays varied by analyte and across two plates, with the following ranges (pg/mL): IL-6: 0.0947–820; IL-5: 0.188–881; TLe2: 74.2–96,900; MIP-1β: 0.169–594; MIP-1α: 0.0403–618; MCP-1: 0.133–397; MDC: 1.31–4,650; SAA: 24.1–242000 and Tau: 368–3630000fg/ml. Each assay was performed in duplicate, and plates were analyzed on a MESO QuickPlex SQ 120 instrument.

### Data Analysis

Raw data were analyzed using TraceFinder 5.1 (Thermo). Metabolite identification and annotation required at least two ions (target + confirming) and a unique retention time that corresponded to the ions and retention time of a reference standard previously determined in-house. A pooled-sample generated prior to derivatization was analyzed at the beginning, at a set interval during, and the end the analytical run to correct peak intensities using the NOREVA tool^52^. NOREVA corrected data were then normalized to the total signal per sample to control for extraction, derivatization, and/or loading effects. Acquired LC-MS data were processed by Thermo Scientific TraceFinder 4.1 software, and metabolites were identified based on the University of Iowa Metabolomics Core facility standard-confirmed, inhouse library. Analyte signal was corrected by normalizing to the deuterated analyte signal and the signal obtained from processing blank (PB) was subtracted. MSD Data acquisition and analysis were conducted with MSD Discovery Workbench software version 4.0, ensuring high precision and reproducibility across all measurements.

### Statistical Analysis

Pre- to post-intervention changes in the outcomes (cerebral blood flow, metabolic outcomes, CBC and cytokine levels) were analyzed on the natural logarithmic scale using paired t-tests. The software of the MIXED procedure of SAS version 9.4 (SAS Institute Inc., Cary, NC) was utilized to conduct the paired t-tests. Paired t-tests were utilized to test the null hypothesis that the mean pre-to post-intervention change in the outcome for each group (e.g., Δ log_e_ [global cerebral blood flow]) was equal to zero, versus the two-sided alternative hypothesis that the mean change was not equal to zero. The null hypotheses were tested separately for APOE4 carriers and non-carriers. All null hypotheses were tested at a two-sided significance level of 0.05.

## Figures and Tables

**Table 1.** Inclusion and exclusion criteria

Inclusion Criteria	Exclusion Criteria
Age 45–65 years old	Diagnosis of mild cognitive impairment (MCI) or dementia, including Alzheimer's disease
Male or Female, all ethnic groups	BMI ≥ 35 (based on MRI feasibility)
MoCA score ≥ 26	Diabetes (HbA1c ≥ 6.5% or antidiabetic medications)
CDR Dementia Instrument = 0	History of skin ulcers or poor wound healing
Carrier Cohort: APOE4 homozygous or heterozygous as determined by buccal swab	Current tobacco or illicit drug use or alcohol abuse (defined as ≥ 4 per day or ≥ 14 per week for men and ≥ 3 per day or ≥ 7 per week for women; per NIAAA guidelines)
Non-carrier cohort: no APOE4 gene identified, as determined using buccal swab; includes APOE2 and 3	Use of anti-platelet or anti-coagulant medications other than aspirin
	Current medications that affect cytochrome P450 3A4 (CYP3A4)
	Immunosuppressant therapy within the last year
	Chemotherapy or radiation treatment within the last year
	Current or chronic history of liver or kidney disease or known hepatic or biliary abnormalities
	Untreated hypertriglyceridemia (fasting triglycerides < 300 mg/dL)
	Current or significant history of pulmonary disease
	Chronic heart failure
	Pregnancy or lactation
	Recent history (past 6 months) of myocardial infarction, active coronary artery disease, intestinal disorders, stroke, or transient ischemic attack
	Poorly controlled blood pressure (systolic BP>160 or diastolic BP>100 mmHg)
	Active inflammatory, COVID–19, autoimmune, infectious, hepatic, gastrointestinal, malignant, and/or severe mental illness
	History of, or MRI, or CT positive for, any space occupying brain lesion, including mass effect of abnormal intracranial pressure
	Organ transplant recipients
	History of stroke
	History of ruptured intracranial aneurysm
	Any condition for which an MRI procedure is contraindicated. Some examples include metallic material in the body, such as pacemakers, metallic clips, etc.
	Likelihood of claustrophobia

**Table 2.** Demographic and baseline characteristics of APOE4 carriers and non-carriers.

	APOE4 carriers E4(+)	Non-carriers E4(−)
**N (F:M)**	9 (4:5)	14 (7:7)
**Age (years)**	53.2 ± 7.6	54.6 ± 6.2
**Ethnicity**	Caucasian (9)	Caucasian (14)
**BMI**	28.9 ± 3.4	26.7 ± 4.1
**MoCA**	28.2 ± 1.3	27.9 ± 1.5
**Blood glucose (mg/dL)**	94.6 ± 11.6	95.6 ± 12.8
**HbAlc (%)**	5.4 ± 0.3	5.3 ± 0.3

Data are presented as mean ± standard deviation. BMI: Body Mass Index, MoCA: Montreal Cognitive Assessment and F:M: Female-to-Male ratio.

**Table 3.** Hematological data before and after the Rapamycin administration.

Groups	APOE4 carriers (E4(+))			Non-carriers (E4(−))		
Timepoint	Pre	Post	P-value	Pre	Post	P-value
**Rapamycin level (ng/mL)**	<1.0	4.0(1.4)	< 0.05*	<1.0	3.8(2.0)	< 0.05*
**BMI**	28.9 (3.4)	28.7 (3.2)	> 0.05	26.7 (4.1)	26.8 (4.2)	> 0.05
**WBC x10(9)/L**	6.4 (1.4)	6.0 (1.5)	0.2146	5.6 (1.4)	5.7 (1.7)	0.9470
**RBC x10(12)/L**	4.8 (0.5)	4.9 (0.5)	0.1895	4.9 (0.5)	4.9 (0.6)	0.6120
**HGB g/dL**	14.3 (1.2)	14.5 (1.5)	0.5483	14.2 (1.2)	14.0 (1.4)	0.1655
**MCV fL**	89.5 (4.2)	88.9 (4.2)	0.1397	87.2 (6.2)	86.2 (5.9)	0.0263*
**MCH pg**	30.0 (1.5)	29.7 (1.6)	0.2100	29.1 (2.4)	28.7 (2.4)	0.0122*
**RDW SD fL**	40.9 (2.3)	39.2 (2.5)	0.0092*	41.6 (3.5)	39.3 (2.9)	0.0002*
**RDW CV %**	12.5 (0.5)	12.0 (0.3)	0.0007*	13.1 (1.1)	12.5 (0.9)	0.0002*
**PLT x10(9)/L**	237.5 (37.7)	252.5 (46.9)	0.0722	238.8 (61.6)	245.9 (63.3)	0.4081
**MPV**	10.0 (0.9)	9.8 (0.9)	0.3733	10.5 (0.9)	10.2 (0.9)	0.0036*
**% Immature Granulocytes**	0.5 (0.3)	0.2 (0.1)	0.0429*	0.4 (0.3)	3.8 (12.7)	0.1532
**% Lymphocytes**	34.2 (6.4)	32.5 (6.8)	0.2419	32.3 (6.0)	30.0 (10.1)	0.3211
**Abs Lymphocytes x10(9)/L**	2.2 (0.7)	2.0 (0.6)	0.0006*	1.8 (0.5)	1.7 (0.6)	0.2851
**Abs Basophils x10(9)/L**	0.04 (0.01)	0.03 (0.01)	0.0276*	0.04 (0.02)	0.03 (0.02)	0.0409*
**Sodium (mmol/L)**	139.4 (1.4)	139.6 (1.4)	0.6974	140.2 (1.6)	140.2 (2.0)	0.9926
**Potassium (mmol/L)**	4.3 (0.2)	4.2 (0.3)	0.6495	4.3 (0.5)	4.1 (0.4)	0.0254*
**Anion gap (mmol/L)**	13.6 (2.1)	13.2 (2.1)	0.5654	12.9 (1.3)	13.7 (1.4)	0.0271*
**Glucose (mg/dL)**	94.6 (11.6)	93.4 (6.4)	0.7200	95.6 (12.8)	94.6 (12.1)	0.5768
**Creatinine (mg/dL)**	1.0 (0.2)	1.0 (0.2)	0.1214	0.9 (0.2)	0.9 (0.2)	0.0611
**Total Protein (g/dL)**	7.2 (0.4)	7.2 (0.6)	0.8708	6.9 (0.4)	7.0 (0.3)	0.0414*
**Albumin (g/dL)**	4.3 (0.1)	4.2 (0.3)	0.1492	4.4 (0.1)	4.3 (0.1)	0.0844
**Total Bilirubin (mg/dL)**	0.6 (0.2)	0.4 (0.2)	0.1136	0.5 (0.2)	0.5 (0.2)	0.0766
**Alkaline Phosphatase (units/L)**	71.9 (10.2)	77.5 (12.7)	0.0601	84.2 (32.8)	92.2 (38.4)	0.0445*
**AST (units/L)**	19.4 (4.7)	20.0 (4.9)	0.6752	21.8 (7.6)	22.4 (5.8)	0.5015
**ALT (units/L)**	22.6 (10.5)	21.6 (7.6)	0.7019	24.2 (13.4)	27.3 (18.8)	0.3433
**Triglycerides (mg/dL)**	133.9 (63.8)	135.3 (58.2)	0.7772	89.3 (36.7)	95.9 (45.7)	0.3262
**HbA1c %**	5.4 (0.3)	5.5 (0.3)	0.0168	5.3 (0.3)	5.4 (0.3)	0.0779

Data presented as mean (standard deviation) for each parameter. **WBC**: White Blood Cell count; **RBC**:Red Blood Cell count; **HGB**: Hemoglobin; **MCV**: Mean Corpuscular Volume; **MCH**: Mean CorpuscularHemoglobin; **RDW**: Red Cell Distribution Width; **PLT**: Platelet count **MPV**: Mean Platelet Volume; **AST**: Aspartate Aminotransferase; **ALT**: Alanine Aminotransferase.

**Table 4.** Comparison of key changes induced by Rapamycin in the APOE4 carriers vs. non-carriers.

	APOE4 carriers (E4(+))	Non-carriers (E4(−))
Cerebral blood flow (CBF)	Significantly Increased (potentially driven by females)	No change
Plasma metabolomics	Increased in G3P, Cinnamate and Cadaverine Decreased in Thymine	Increased in KIV, Glycerol and Linoleate Decreased in GABA, G6P and Inositol
Inflammatory makers	Decreased in MCP-1 and Tle-2	Increased in IL-5 and MDC Decreased in MIP-1α and MIP -1β
Plasma SAA (Aβ) and Tau	No change	No change
Short chain fatty acid (SCFA)	Increased in Butyrate and Propionate	No change
Gut microbiome	Significant differences in diversity at the baseline between the two groups Rapamycin enriched *Bacteroides salyersiae, B. stercoris,* and *Butyncicoccus* sp. in E4{+) Rapamycin enriched *ntestinimonas* sp. and an unclassified *Ruminococcaceae* genus in E4(−)	
**Similar changes in both groups**	Decreased in DOPA Increased in IL-6 Decreased in Red cell distribution width (RDW)	

## Data Availability

The minimum datasets necessary to interpret, verify and extend the research in the article are available within the paper. The trial was registered on ClinicalTrials.gov: 05386914.
